# Charges for Initial Visits for Uninsured Patients at Musculoskeletal Urgent Care Centers in the US

**DOI:** 10.1001/jamanetworkopen.2022.9968

**Published:** 2022-05-03

**Authors:** Laurie C. Yousman, Walter R. Hsiang, Akshay Khunte, Michael Najem, Grace Jin, Alison Mosier-Mills, Siddharth Jain, Daniel Wiznia

**Affiliations:** 1 Yale School of Medicine, New Haven, Connecticut; 2 Department of Urology, University of California, San Francisco; 3 Kaiser Permanente Bernard J. Tyson School of Medicine, Pasadena, California; 4 Graduate Institute of International and Development Studies, Genève, Switzerland; 5 Harvard Medical School, Boston, Massachusetts; 6 Department of Orthopaedics and Rehabilitation, Yale School of Medicine, New Haven, Connecticut

## Abstract

**Importance:**

In recent years, specialized musculoskeletal urgent care centers (MUCCs) have opened across the US. Uninsured patients may increasingly turn to these orthopedic-specific urgent care centers as a lower-cost alternative to emergency department or general urgent care center visits.

**Objective:**

To assess out-of-pocket costs and factors associated with these costs at MUCCs for uninsured and underinsured patients in the US.

**Design, Setting, and Participants:**

In this survey study, a national secret shopper survey was conducted in June 2019. Clinics identified as MUCCs in 50 states were contacted by telephone by investigators using a standardized script and posing as uninsured patients seeking information on the out-of-pocket charge for a new patient visit.

**Exposures:**

State Medicaid expansion status, clinic Medicaid acceptance status, state Medicaid reimbursement rate, median income per zip code, and clinic region.

**Main Outcomes and Measures:**

The primary outcome was each clinic's out-of-pocket charge for a level 3 visit, defined as a new patient office visit requiring medical decision-making of low complexity. Linear regression was used to examine correlations of price with clinic policy against accepting Medicaid, median income per zip code, and Medicaid reimbursement for a level 3 visit.

**Results:**

Of 565 MUCCs identified, 558 MUCCs were able to be contacted (98.8%); 536 of the 558 MUCCs (96.1%) disclosed a new patient visit out-of-pocket charge. Of those, 313 (58.4%) accepted Medicaid insurance and 326 (60.8%) were located in states with expanded Medicaid at the time of the survey. The mean (SD) price of a visit to an MUCC was $250 ($110). Clinic policy against accepting Medicaid (β, 22.91; 95% CI, 12.57-33.25; *P* < .001), higher median income per zip code (β, 0.00056; 95% CI, 0.00020-0.00092; *P* = .003), and increased Medicaid reimbursement for a level 3 visit (β, 0.737; 95% CI, 0.158-1.316; *P* = .01) were positively correlated with visit price. The overall regression was statistically significance (*R*^2^ = 0.084; *P* < .001).

**Conclusions and Relevance:**

In this survey study, MUCCs charged a mean price of $250 for a new patient visit. Medicaid acceptance policy, median income per zip code, and Medicaid reimbursement for a level 3 visit were associated with differences in out-of-pocket charges. These findings suggest that accessibility to orthopedic urgent care at MUCCs may be limited for underinsured and uninsured patients.

## Key Points

**Question:**

What factors are associated with out-of-pocket charges for a first visit to a musculoskeletal urgent care center for uninsured and underinsured patients in the US?

**Findings:**

In this nationwide survey study of 565 musculoskeletal urgent care centers in the US, lack of state expanded Medicaid, lack of Medicaid acceptance, and higher median income per zip code were associated with higher out-of-pocket charges for uninsured patients.

**Meaning:**

The findings suggest that clinic policies related to out-of-pocket charges may indicate a profit-driven center model creating disincentives for the treatment of uninsured patients.

## Teaser

This survey study assesses out-of-pocket costs and factors associated with these costs for a first visit to a musculoskeletal urgent care center for uninsured and underinsured patients in the US.

## Introduction

Musculoskeletal urgent care centers (MUCCs), alongside general urgent care centers, have rapidly emerged across the US, ostensibly as an alternative to emergency departments (EDs) and general urgent care centers. Although these orthopedic-specific urgent care centers have had increased use, their effect on access to musculoskeletal care is unclear. There is conflicting research on the benefits and drawbacks of MUCCs, with some literature demonstrating a potential reduction in both cost of care and wait times and other studies questioning long-term savings associated with use of MUCCs.^1,2^ Regardless, the increased access to orthopedic specialists that an MUCC provides is not equally available to all patients. In a recent study of 29 MUCCs in Connecticut, most clinics either denied patients with Medicaid insurance or implemented barriers to care that were not experienced by their privately insured counterparts.^3^ In a comprehensive national survey of all MUCCs in the US, half of all centers surveyed did not accept Medicaid insurance.^4^ Given the large number of uninsured patients in the US, it is important to characterize the charges that patients without insurance coverage would incur if they were to seek urgent orthopedic care at these centers. Furthermore, freestanding EDs providing urgent care services have been established in areas with a more profitable payer mix.^5^ To our knowledge, no studies have characterized the payment practices of MUCCs for uninsured patients. In addition, identifying the factors associated with charges for care is imperative to understanding how the relatively new and largely unexplored MUCCs fit into the broader system of health care in the US. In conducting this survey, we assessed out-of-pocket costs and factors associated with these costs at MUCCs. We hypothesized that Medicaid acceptance would be associated with a reduction in out-of-pocket charges.

## Methods

This survey study was reviewed and given institutional review board exemption by the Yale University institutional review board, with a waiver of informed consent because this was non–human participant research. A secret shopper method was used to obtain the visit price at an MUCC and the factors associated with this price. This study followed the American Association for Public Opinion Research (AAPOR) reporting guideline. An online search was conducted in June 2019 using multiple query methods to identify a comprehensive list of MUCCs in 50 states. We defined an MUCC as a walk-in clinic dedicated to assessing urgent orthopedic needs outside a hospital ED or freestanding ED. First, Google Maps was used to perform a search in each state for the following terms: *orthopedic urgent care*, *orthopedic and sports injury walk-in clinic*, and *musculoskeletal urgent care*. Second, we accessed the urgent care directory of Solv,^6^ an online booking website for physician offices and urgent care centers, to generate another list of MUCCs per state. Finally, we used the urgent care center finder of the Urgent Care Association,^7^ an urgent care membership organization of nearly 4000 urgent care centers, to identify additional MUCCs in each state. We excluded general urgent care centers or physician offices that offered orthopedic services.

**Figure 1.**
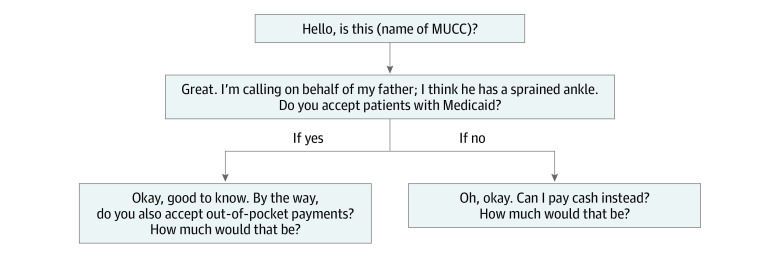
Standardized Call Script MUCC indicates musculoskeletal urgent care center.

In June 2019, researchers attempted to contact each MUCC by telephone (Figure 1). They used a standardized script and posed as uninsured patients who had recently sprained their ankle and requested the basic visit price without charges for radiographic services or laboratory examinations. If an office refused or was unable to provide a visit price, they were called back on a different day. If they were unable to provide a visit price after the subsequent call, they were excluded from the data set. Out-of-pocket charges were characterized in all states except for Delaware because only 2 MUCCs were located and neither provided a price. Because all MUCCs surveyed were walk-in clinics, no formal appointment or engagement with a physician or medical staff was scheduled. The primary outcome measure was the visit price at each clinic. If the office could not specify a single value and gave a price range, the mean was taken. In the context of this study, the term *charge* is used to describe the out-of-pocket price for a basic patient visit. Because uninsured patients must pay out of pocket, their personal cost of care was equivalent to the entire charge presented by the MUCC. Researchers also asked whether the MUCC accepted Medicaid insurance.

Individual center and state-level information was collected. Median income per zip code, Medicaid acceptance status, and practice classification (academic affiliation, extension of a private practice or hospital, or unaffiliated) were collected for each MUCC. Practice classification was determined from information found on each MUCC’s website. State-level data were collected on geographic region, Medicaid expansion status, and Medicaid reimbursement for a level 3 new patient visit, defined as a new patient office visit requiring medical decision making of low complexity. State Medicaid expansion status was based on data from the Kaiser Family Foundation.^8^ Level 3 patient visit reimbursement data were located through a manual search on each state’s government Medicaid physician fee schedule website. Income and population statistics and geographic region of MUCCs were based on data from the US Census Bureau.^9^

### Statistical Analysis

**Figure 2.**
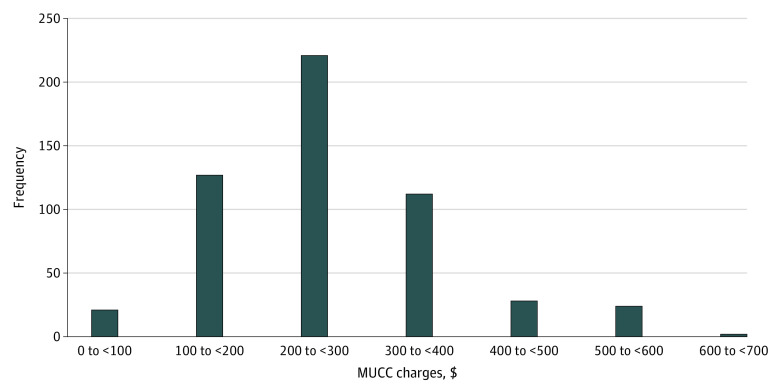
Distribution of Musculoskeletal Urgent Care Center (MUCC) Charges

Statistical analysis was performed using JMP Pro, version 13 (SAS Institute Inc). Proportions of centers were calculated for region, practice classification, Medicaid acceptance status, and location in a Medicaid expansion state. In the preliminary bivariate analysis for each variable of interest, Student *t* tests were used to examine correlations of price with Medicaid acceptance and state Medicaid expansion. One-way analysis of variance was used to examine correlations of price with MUCC region and MUCC classification type. Linear regression was used to examine correlations of price with median income per zip code and Medicaid reimbursement for a level 3 visit. These tests were justified by a relatively normal distribution of charges (Figure 2). The associations of state Medicaid expansion with MUCC charges and Medicaid acceptance were examined using a Student *t* test and an odds ratio, respectively. To control for the effects of multiple variables, multiple regression was performed with significant variables that did not exhibit substantial correlation with another variable. Two-sided *P* <.05 was considered significant.

## Results

Of the 565 MUCCs identified, 558 were able to be contacted (98.8% response rate). Of the 558 MUCCs reached, 536 (96.1%) disclosed a out-of-pocket charge. Among these, 326 (60.8%) were in states with expanded Medicaid, and 210 (39.2%) were in states without Medicaid expansion. Of the 536 MUCCs, 313 (58.4%) accepted Medicaid. A total of 124 centers (23.1%) were located in the Northeast, 69 (12.9%) in the West, 133 (24.8%) in the Midwest, and 210 (39.2%) in the South. A total of 45 centers (8.4%) were classified as academic MUCCs, 30 (5.6%) as nonaffiliated MUCCs, and 461 (86%) as affiliated with a private practice. Table 1 gives the characteristics of the sample.

**Table 1.** Characteristics of MUCCs

Characteristic	MUCCs, No. (%)
Classification	
Academic	45 (8.4)
Extension of a private practice or hospital	461 (86.0)
Nonaffiliated	30 (5.6)
Region	
Northeast	124 (23.1)
West	69 (12.9)
Midwest	133 (24.8)
South	210 (39.2)
MUCC Medicaid expansion status	
Expanded	326 (60.8)
Not expanded	210 (39.2)
Accepts Medicaid	
Yes	313 (58.4)
No	223 (41.6)

Abbreviation: MUCC, musculoskeletal urgent care center.

Across all states, a level 3 new patient visit to an MUCC was found to have a mean (SD) out-of-pocket charge of $250 ($110). Table 2 shows the associations between classifications of the MUCCs in the sample and visit price based on bivariate analysis. Centers that did not accept Medicaid had a greater mean visit price than centers that accepted Medicaid (difference; $49; 95% CI, $31-$67; *P* < .001). In addition, the mean visit price was $27 (95% CI, $17-$36; *P* < .001) greater for MUCCs in states without expanded Medicaid compared with centers in states with expanded Medicaid. Charges for musculoskeletal urgent care varied from region to region, with MUCCs in the South having the greatest mean visit price ($266; 95% CI, $251-$281) and MUCCs in the West having the lowest mean visit price ($236; 95% CI, $210-$262). Louisiana had the greatest out-of-pocket charges, with an mean of $373 (95% CI, $167-$579). South Dakota had the lowest prices, with an mean of $133 (95% CI, $88-$177). No significant difference in mean visit price based on MUCC classification was found.

**Table 2.** Bivariate Analysis of Categorical MUCC Characteristics Associated With Cost of Care

Characteristic	Visit price, mean (95% CI), $	*P* value
Classification of center		
Academic (n = 45)	257 (224-289)	.90
Extension of a private practice or hospital (n = 461)	249 (239-259)	
Nonaffiliated (n = 30)	246 (206-286)	
Region		
Northeast (n = 124)	241 (222-260)	<.048
West (n = 69)	236 (210-262)	
Midwest (n = 133)	238 (220-257)	
South (n = 210)	266 (251-281)	
MUCC Medicaid expansion status		
Expanded (n = 326)	239 (227-251)	<.001
Not expanded (n = 210)	266 (251-281)	
Accepts Medicaid		
Yes (n = 313)	229 (217-241)	<.001
No (n = 223)	278 (264-292)	

Abbreviation: MUCC, musculoskeletal urgent care center.

Multiple linear regression was used to assess whether a variable remained significant after adjustment for the influence of other variables (Table 3). Medicaid acceptance status, clinic region, median income per zip code, and level 3 Medicaid reimbursement rate were used in the fitted regression model. The overall regression was statistically significance (*R*^2^ = 0.084, *P* < .001). Lack of Medicaid acceptance (β = 22.91; 95% CI, 12.57-33.25; *P* < .001), increased median income per zip code (β = 0.00056; 95% CI, 0.00020-0.00092; *P = *.003), and Medicaid reimbursement for a level 3 visit (β, 0.737; 95% CI, 0.158-1.316; *P* = .01) were correlated with increased charges for care.

**Table 3.** Multivariate Analysis of MUCC Characteristics Associated With Cost of Care

Characteristic	β (95% CI)	*P* value
Does not accept Medicaid	22.91 (12.57 to 33.25)	<.001
Median income per zip code	0.00056 (0.00020 to 0.00092)	.003
Medicaid reimbursement for a level 3 visit	0.737 (0.158 to 1.316)	.013
Region		
West	1 [Reference]	NA
Midwest	4.43 (−12.80 to 21.66)	.61
Northeast	−7.69 (−25.53 to 10.14)	.40
South	20.93 (5.35 to 36.51)	.009

Abbreviations: MUCC, musculoskeletal urgent care center; NA, not applicable.

State Medicaid expansion was not associated with an increased likelihood of Medicaid acceptance (odds ratio, 1.19; 95% CI, 0.84-1.69; *P* = .34). Centers in states with expanded Medicaid had a lower mean visit price compared with centers in states without expanded Medicaid (difference, $27; 95% CI $8-$45; *P* = .004).

## Discussion

MUCCs have emerged across the US as potential lower-cost alternatives to EDs, ostensibly to simultaneously reduce charges and increase accessibility of orthopedic care.^1^ In addition, use of MUCCs has increased, as has use of general urgent care centers, with a similar appeal of potential cost-savings. Our study sought to understand the true cost of care for uninsured patients at MUCCs by identifying charges for musculoskeletal urgent care in the US and the factors associated with these charges. To understand whether MUCCs are truly cost-saving institutions, it is necessary to examine the priorities of these clinics and other options for immediate care, including EDs and general urgent care centers.

The existing literature^3,4^ indicates that MUCCs operate on a profit-driven model, having clinic policies in place that ensure a certain payer mix. Therefore, care at MUCCs may be unavailable to patients who are most in need of cost savings: the uninsured and underinsured. One previous study found that MUCCs in Connecticut were located in higher-income neighborhoods.^3^ Multiple studies have confirmed that a high percentage of MUCCs do not accept Medicaid insurance.^3,4^ Urgent care centers are not subject to the Emergency Medical Treatment and Active Labor Act and therefore are legally able to screen patients based on their perceived ability to pay. Our findings suggest that a practice’s policy of Medicaid acceptance, median household income per zip code, and Medicaid reimbursement for a level 3 visit are associated with visit charges at MUCCs. These findings are in line with previous literature^2,3,4^ characterizing MUCCs operating on a profit-based model. Of note, Medicaid insurance acceptance was found to be significantly associated with visit price, with centers not accepting Medicaid generally having higher mean visit prices than those that accepted Medicaid. MUCCs have been shown to be more likely to provide treatment to privately insured patients than to their Medicaid-insured counterparts.^3,4^ Operating under a profit-driven model, centers that did not accept Medicaid prioritized optimizing their payer mix by limiting Medicaid-insured patients. The higher charges at MUCCs not accepting Medicaid, which accounted for 41.6% of the sample, suggest that these centers are more profit-driven than MUCCs that accept Medicaid.

In addition, when comparing an MUCC with an ED, potential surface-level, short-term cost savings may not correspond to true savings over time. Not all urgent orthopedic conditions are appropriate for MUCCs, and individuals with surgical emergencies must go to the ED regardless, in which case the MUCC charges would be additive to ED charges and costs; potential savings would only apply to low-acuity cases. Furthermore, although initial upfront charges for lower-acuity visits to urgent care centers may be lower than those in the ED, these charges do not account for factors like quality of care, care coordination, laboratory and radiology services, and durable medical equipment. One study^2^ from 2021 supported the existing literature’s findings of higher charges at EDs than at urgent care centers. However, that study examined the substitution ratio, defined as the number of urgent care visits that would deter 1 ED visit. The study found a substitution ratio of 37, with 37 urgent care visits replacing a single ED visit. Even if the upfront charge for a low-acuity visit was 10 times lower at an urgent care center, repeated visits would ultimately yield a higher overall cost of care.^2^ In addition, Medicaid patients do not pay out-of-pocket charges for ED visits and therefore do not have a direct financial incentive to seek care at an MUCC.

The most relevant comparison for cost-conscious uninsured patients with nonemergent orthopedic injuries may be between an MUCC and a general urgent care center if the patient is mostly focused on immediate savings. In the sample of nearly all MUCCs in the US in our study, we showed that the mean price of a basic visit to an MUCC was $250. In contrast, in the existing literature, the mean price of a basic visit to a general urgent care center was approximately $100 less, with estimates from different studies being $149, $162, and $178.^2,10,11^ Estimates for both MUCCs and general urgent care centers were for a basic visit only, not for any accompanying laboratory or specialty services. One may assume that owing to the specialization of centers marketed as MUCCs, they would provide unique orthopedic services that justify their increased cost. However, many of the largest general urgent care center chains provide orthopedic services, including x-rays, treatment of sprains, and setting of fractures.^12,13,14^ To our knowledge, no studies have assessed outcomes or cost savings associated with treatment at MUCCs compared with general urgent care centers.

### Limitations

This study has several limitations. First, given the lack of a centralized database for all MUCCs, we conducted a manual online search, which may not have captured all centers in the US. However, we worked to mitigate this limitation by combining search results from 3 different methods. In addition, our findings represent characteristics and charges at MUCCs at a single point in time, June 2019. The recent increases in MUCCs and evolving national health care policy may influence whether our findings hold true in the future. The study also separated states based on Medicaid expansion status and centers based on acceptance of Medicaid but could not account for possible variations in Medicaid policies among different states, which might influence the visit price of MUCCs.

## Conclusions

In this survey study, lack of acceptance of Medicaid insurance, higher median income per zip code, and increased Medicaid reimbursement for a level 3 visit were associated with higher charges at MUCCs, suggesting a profit-driven model designed to optimize payer mix. In addition, compared with visit prices at general urgent care centers, visit prices at MUCCs were found to be higher, with outcome or cost-saving justification for this increase in cost not evident in the literature to date. Future studies should investigate charges for follow-up visits, surgical intervention, and longer-term care after initial interventions at MUCCs compared with EDs and general urgent care centers and should analyze outcomes and quality for patients with orthopedic conditions at MUCCs compared with general urgent care centers.
